# Education Moderates the Association of Probable REM Sleep Behavior Disorder With Cognitive and Motor Impairments in Community-Dwelling Older People

**DOI:** 10.3389/fneur.2020.00109

**Published:** 2020-02-28

**Authors:** Meijie Chen, Jie Chen, Xitong Xu, Fangwei Qiao, Xue Wang, Shaozhen Ji, Zhuqin Gu, Jagadish K. Chhetri, Piu Chan

**Affiliations:** ^1^Department of Neurobiology, Neurology and Geriatrics, Xuanwu Hospital of Capital Medical University, Beijing Institute of Geriatrics, Beijing, China; ^2^Clinical Center for Parkinson's Disease, Advanced Innovation Center for Human Brain Protection, Capital Medical University, Beijing, China; ^3^Key Laboratory for Neurodegenerative Disease of the Ministry of Education, Beijing Key Laboratory for Parkinson's Disease, Parkinson Disease Center of Beijing Institute for Brain Disorders, Beijing, China; ^4^National Clinical Research Center for Geriatric Disorders, Beijing, China

**Keywords:** rapid eye movement (REM) sleep behavior disorder (RBD), cognition, motor, gait, reserve, education

## Abstract

**Objectives:** To investigate the relationship between probable rapid eye movement (REM) sleep behavior disorder (pRBD) and cognitive/motor impairments in a community-dwelling population and explore the moderating effects of education.

**Methods:** In this cross-sectional study of the Beijing Longitudinal Study of Aging II (BLSA II), 4,477 subjects (≥55 years) fulfilled the inclusion criteria. pRBD was determined by the RBD Questionnaire–Hong Kong (RBDQ-HK). Mini-Mental State Examination (MMSE) and Montreal Cognitive Assessment (MoCA) were used to test the global cognitive performance. Walking speed was used to measure motor function. Logistic regression was performed to assess the relationship between pRBD and cognitive/motor impairments and the moderating effects of education.

**Results:** There were 147 participants (3.3%) with pRBD. Participants with pRBD showed increased risks for cognitive impairment [odds ratio (OR) = 1.88, 95% CI 1.24–2.85, *p* = 0.003], decreased gait speed (OR = 1.43, 95% CI 1.02–2.01, *p* = 0.03), but not for mild cognitive impairment (MCI) (measured by MoCA: OR = 1.01, 95% CI 0.68–1.50, *p* = 0.95; measured by MMSE: OR = 0.90, 95% CI 0.59–1.37, *p* = 0.62). Education modified the effect of pRBD on MCI (measured by MoCA: *p* < 0.001; measured by MMSE: *p* = 0.061) and gait speed (*p* = 0.008).

**Conclusions:** Our findings suggest that pRBD increases the risk of cognitive/motor impairments for a community-dwelling older population, and education could alleviate the negative effects. These findings implicate that education may have beneficial effects on delaying the onset of cognitive/motor decline in pRBD subjects.

## Introduction

Rapid eye movement (REM) sleep behavior disorder (RBD) is a parasomnia characterized by vivid dreams, dream-enacting behaviors, and loss of REM sleep atonia (1). Idiopathic RBD (iRBD) often occurs in individuals aged 50 years and above and is known to present a strong association with α-synucleinopathy (2, 3). Over 70% of patients with iRBD were reported to develop parkinsonism or dementia after a 12 year follow-up (4). Thus, such long prodromal interval provides a unique opportunity to explore disease-modifying therapies to potentially prevent the development of much severe neurological conditions such as parkinsonism and dementia (5).

Diagnosis of RBD requires video polysomnography (vPSG), which is less convenient for large-scale studies given the high expenses and lack of trained specialists. However, a diagnosis of probable RBD (pRBD) can be made much conveniently using validated questionnaires with good reliability and diagnostic accuracy (6). Screening for pRBD in the general population could enable early identification of RBD patients and allow us to investigate the moderating factors accordingly. Very few studies have reported an association between pRBD and impaired motor/cognitive functions in the general population (7–9). In addition, the moderating factors associated with pRBD still remain to be investigated.

Education attainment is a modifiable factor known to moderate cognitive and motor performances in old age (10, 11). Individuals with higher education levels are known to have a higher cognitive reserve, which enables them to withstand pathological changes (12). For instance, epidemiological studies suggest that individuals with a higher level of education have lower risks of dementia (13) and show better cognitive performance even in old age (14). Recently, a longitudinal study conducted in patients with Parkinson's disease (PD) suggested that higher education was associated with not only better baseline cognitive performance but also better baseline motor function (15). The mechanism by which education influences PD is not very clear. Limited studies suggested that it may be related to the brain white matter integrity (16) and brain metabolism (17). Since RBD represents the prodromal stage of α-synucleinopathy including PD, there is a possibility that education has a beneficial effect on motor and cognitive functions not only when the disease is overt but also in prodromal stages such as RBD individuals. Understanding the effects of education in subjects with pRBD may provide important implications for early interventions designed to delay the clinical onset of motor and cognitive decline in α-synucleinopathies.

The aim of this study was to investigate the potential relationship between pRBD and motor/cognitive functions in a community-based population and explore the influence of education on this relationship.

## Methods

### Study Sample

**Figure 1:**
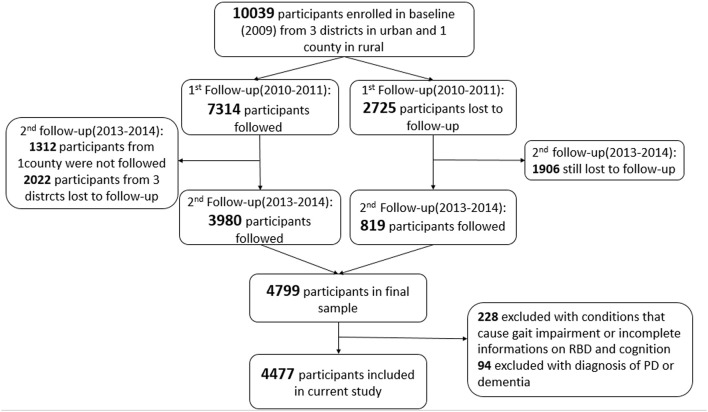
Flowchart of the study.

The Beijing Longitudinal Study of Aging II (BLSA II) is a community-based prospective cohort study, which was launched in 2009 in Beijing, China. The original study consisted of 10,039 participants aged 55 years and older from three urban districts and one rural county. Details of the study have been published elsewhere (18, 19). The participants in our study were enrolled from the second follow-up of the BLSA II study. A total of 4,799 participants were followed during 2013–2014 from three urban districts. Cognitive assessments and RBD evaluations were performed by trained clinicians (Figure 1). We excluded 228 participants (4.75%) with preexisting conditions that could cause gait impairment or with incomplete information on cognition, gait, and RBD. We further excluded 94 (1.96%) participants with a pre-diagnosis of PD or dementia. Finally, a total of 4,477 participants (93.3%) were included in our current study.

The study was approved by the Research Ethics Committee of Xuanwu Hospital of Capital Medical University [Number: Xuanwu (2011)27], and each participant provided written informed consent.

### Assessment of Cognitive Function

Cognitive function was assessed using the Mini-Mental State Examination (MMSE) and the Montreal Cognitive Assessment (MoCA). Subjects with MMSE score <24 were considered as having cognitive impairment, as suggested by a previous study (20). Two different definitions were applied to diagnose mild cognitive impairment (MCI) in all subjects. One criteria is MoCA < 26 (21), and the other is MMSE < 28 (22) for participants with normal MMSE scores (i.e., MMSE ≥ 24) to better evaluate cognitive status.

### Assessment of Motor Function

Motor function was assessed by walking speed derived from the Fried's criteria of frailty scale (23). Walking speed was evaluated using the time spent through a 15-ft (4.6 m) walking test. Decreased walking speed was defined as the slowest 20% of the population: ≥6 s for males with height > 173 cm, ≥ 7 s for males with height ≤ 173 cm, ≥6 s for females with height > 159 cm, and ≥7 s for females with height ≤ 159 cm (23).

### Diagnosis of Probable Rapid Eye Movement Sleep Behavior Disorder

pRBD was determined by the REM Sleep Behavior Disorder Questionnaire–Hong Kong (RBDQ-HK) (24). The RBDQ-HK is composed of 13 items related to RBD clinical features and evaluates the presence, frequency, and severity of RBD symptoms. An RBDQ-HK score of 19 or more was considered to be pRBD.

### Assessment of Education and Other Covariates

Educational level was classified into three categories: (a) low level of education for illiteracy or primary school graduates; (b) middle level of education for middle school graduates; (c) high level of education for high school graduates or above.

The presence of medical conditions such as hypertension, diabetes, hyperlipemia, and stroke was based on a physician's diagnosis during the follow-up study or a self-reported history of a diagnosed condition. Depression was defined as a score of 11 or more in the Geriatric Depression Scale-30 (GDS-30).

### Statistical Analyses

Participants' characteristics were described and compared according to their RBD status. For continuous variables, one-way analysis of variance and Mann–Whitney rank tests were used to assess the significance of differences between the no-pRBD and pRBD groups. For binary variables, chi-square test was used for the analysis. Logistic regression models were used to evaluate the relationship between pRBD and cognitive/motor functions and the interaction effect between education and pRBD. Model 1 was adjusted only for the demographic variables including age, gender, and educational level. The variable of gender was excluded while examining motor function because it had been already considered when defining reduced walking speed. Model 2 was additionally adjusted for medical conditions such as hypertension, diabetes, hyperlipemia, and stroke (given the association of vascular risk factors with cognitive and motor dysfunctions). In Model 3, cognitive function was additionally adjusted for depression, and motor function was adjusted for depression and MMSE (in addition to all other covariates). All statistical tests were two-sided, and significance was set at *p* < 0.05. SPSS 19.0 (SPSS Inc., Chicago, IL, United States) was used for statistical analyses.

## Results

### Baseline Characteristics of Participants With and Without Probable Rapid Eye Movement Sleep Behavior Disorder

Table 1 shows the characteristics of participants based on pRBD status. A total of 147 participants (3.3%) had pRBD determined by the cutoff value of RBDQ-HK. Participants with hyperlipemia, depression, decreased gait speed, and cognitive impairment were observed to have higher risks of pRBD than subjects without these conditions. There was no significant difference on age, gender, educational level, hypertension, diabetes, and stroke between participants with and without pRBD.

**Table 1:** Characteristics of participants with and without pRBD.

	**No pRBD** **(*N* = 4,330)**	**With pRBD** **(*N* = 147)**	***P*-value**
Age			0.72
Mean (SD)	74.98 (6.71)	74.78 (6.88)	
Gender			0.73
Male	1,661 (38.4%)	54 (36.7%)	
Female	2,669 (61.6%)	93 (63.3%)	
Educational level			0.31
Low	1,489 (34.4%)	56 (38.1%)	
Middle	1,314 (30.3%)	36 (24.5%)	
High	1,527 (35.3%)	55 (37.4%)	
Hypertension			0.09
No	1,569 (36.2%)	41 (27.9%)	
Yes	2,748 (63.5%)	105 (71.4%)	
Unknown	13 (0.3%)	1 (0.7%)	
Diabetes			0.94
No	3,072 (70.9%)	104 (70.7%)	
Yes	1,217 (28.1%)	42 (28.6%)	
Unknown	41 (0.9%)	1 (0.7%)	
Hyperlipemia			<0.001^***^
No	3,371 (77.9%)	78 (53.1%)	
Yes	937 (21.6%)	68 (46.3%)	
Unknown	22 (0.5%)	1 (0.7%)	
Stroke			0.20
No	3,926 (90.7%)	130 (88.4%)	
Yes	386 (8.9%)	15 (10.2%)	
Unknown	18 (0.4%)	2 (1.4%)	
Depression			<0.001^***^
No	3,904 (90.2%)	104 (70.7%)	
Yes	426 (9.8%)	43 (29.3%)	
Decreased gait speed			0.025^*^
No	2,696 (62.3%)	78 (53.1%)	
Yes	1,634 (37.7%)	69 (46.9%)	
Cognitive impairment (defined as MMSE <24)			0.004^**^
No	3,641 (84.1%)	110 (74.8%)	
Yes	689 (15.9%)	37 (25.2%)	
MCI^†^ (defined as MoCA <26)			0.85
No	2,153 (59.1%)	64 (58.2%)	
Yes	1,488 (40.9%)	46 (41.8%)	
MCI^†^ (defined as MMSE <28)			0.61
No	2,390 (65.6%)	75 (68.2%)	
Yes	1,251 (34.4%)	35 (31.8%)	
MMSE score			0.07
Mean (SD)	26.92 (3.69)	26.06 (4.66)	
MoCA score			0.03^*^
Mean (SD)	23.58 (5.70)	22.09 (7.06)	

† *Only for participants with normal MMSE scores (MMSE ≥ 24)*.

*SD, standard deviation; pRBD, probable rapid eye movement sleep behavior disorder; MMSE, Mini-Mental State Examination; MoCA, Montreal Cognitive Assessment; MCI, mild cognitive impairment*.

Significance was set at p < 0.05. Significant differences are highlighted by

* *p < 0.05*,

** p < 0.01, and

*** *p < 0.001*.

### Association Between Probable Rapid Eye Movement Sleep Behavior Disorder and Cognitive/Motor Function

Table 2 shows the relationship between pRBD and cognitive/motor impairments. Participants with pRBD had higher risks of cognitive impairment when adjusted for multiple potential variables including age, gender, educational level, hypertension, diabetes, hyperlipemia, stroke, and depression. For participants with MMSE ≥ 24, the association of pRBD with MCI (measured by MMSE and MoCA scores) was statistically insignificant. Participants with pRBD had higher risks of decreased gait speed when adjusted for age, educational level, hypertension, diabetes, hyperlipemia, stroke, depression, and MMSE score.

**Table 2:** Logistic regression models examining the relationship between pRBD and cognitive performance and motor ability.

	**Model 1**	**Model 2**	**Model 3**
**Association with cognitive impairment (MMSE** **<** **24) for pRBD**			
No. (%) with pRBD	147 (3.3%)	147 (3.3%)	147 (3.3%)
OR (95% CI)	1.884 (1.250–2.840)	1.884 (1.248–2.842)	1.883 (1.243–2.853)
*P*-value	0.002^**^	0.003^**^	0.003^**^
**Association with MCI (MoCA** **<** **26) for pRBD**^**†**^			
No. (%) with pRBD	110 (2.9%)	110 (2.9%)	110 (2.9%)
OR (95% CI)	1.073 (0.727–1.583)	1.086 (0.734–1.606)	1.012 (0.682–1.501)
*P*-value	0.724	0.680	0.953
**Association with MCI (MMSE** **<** **28) for pRBD**^**†**^			
No. (%) with pRBD	110 (2.9%)	110 (2.9%)	110 (2.9%)
OR (95% CI)	0.926 (0.612–1.402)	0.969 (0.638–1.472)	0.900 (0.590–1.372)
*P*-value	0.717	0.882	0.624
**Association with decreased gait speed for pRBD**			
No. (%) with pRBD	147 (3.3%)	147 (3.3%)	147 (3.3%)
OR (95% CI)	1.480 (1.060–2.067)	1.474 (1.055–2.060)	1.431 (1.020–2.007)
*P*-value	0.021^*^	0.023^*^	0.038^*^

† *Only suitable for participants with normal MMSE scores*.

*OR, odds ratio; CI, confidence interval; MoCA, Montreal Cognitive Assessment; MCI, mild cognitive impairment; pRBD, probable rapid eye movement sleep behavior disorder; MMSE, Mini-Mental State Examination*.

Significance was set at p = 0.05. Significant differences are highlighted by

* p < 0.05 and

** *p < 0.01*.

*Model 1: adjusted for age, gender, educational level for cognitive performance; adjusted for age, educational level for motor function*.

*Model 2: adjusted for age, gender, educational level, hypertension, diabetes, hyperlipemia and stroke for cognitive performance; adjusted for age, educational level, hypertension, diabetes, hyperlipemia, and stroke for motor function*.

*Model 3: adjusted for age, gender, educational level, hypertension, diabetes, hyperlipemia, stroke, and depression for cognitive performance; adjusted for age, educational level, hypertension, diabetes, hyperlipemia, stroke, depression, and MMSE score for motor function*.

### Analysis of the Moderating Effect of Education on the Association Between Probable Rapid Eye Movement Sleep Behavior Disorder and Cognitive/Motor Function

To understand the interaction effect between pRBD and education, a separate analysis stratified by different educational levels was performed (Table 3). pRBD was found to increase the risk of MCI (measured with MoCA score) in participants with a low educational level but not in participants with middle and high educational levels (P for interaction < 0.001). When MCI was defined by MMSE score of >24 and <28, there was a tendency of low risk of MCI in participants with middle and high education compared with those with low education (*P* for interaction = 0.061). In addition, pRBD increased the risk of decreased gait speed in participants with low education but not in participants with middle and high education. The interaction effect between pRBD and education on gait speed was significant (*P* for interaction = 0.008). No significant interaction was seen between pRBD and education on cognitive impairment (*P* for interaction = 0.701).

**Table 3:** Logistic regression models examining the relationship between pRBD and cognitive/motor function and the moderating effect of education.

	**No pRBD**		**With pRBD**			
**Cognitive/motor function**	**No. of participants**	**No. of events**	**No. of participants**	**No. of events**	**OR[Table-fn TN8] (95% CI)**	**P for interaction**
**Cognitive impairment (MMSE** **<** **24)**						
Low education	1,489	429 (28.8%)	56	24 (42.9%)	1.938 (1.113–3.375)	
Middle education	1,314	158 (12.0%)	36	8 (22.2%)	2.101 (0.907–4.864)	0.701
High education	1,527	102 (6.7%)	55	5 (9.1%)	1.519 (0.582–3.966)	
**MCI (defined as MoCA** **<** **26)**^**†**^						
Low education	1,060	543 (51.2%)	32	25 (78.1%)	3.393 (1.451–7.938)	
Middle education	1,156	438 (37.9%)	28	11 (39.3%)	1.089 (0.501–2.366)	<0.001^***^
High education	1,425	507 (35.6%)	50	10 (20.0%)	0.478 (0.236–0.968)	
**MCI (defined as MMSE** **<** **28)**^**†**^						
Low education	1,060	478 (45.1%)	32	20 (62.5%)	2.007 (0.954–4.222)	
Middle education	1,156	391 (33.8%)	28	4 (14.3%)	0.353 (0.120–1.035)	0.061
High education	1,425	382 (26.8%)	50	11 (22.0%)	0.749 (0.372–1.507)	
**Decreased gait speed**						
Low education	1,489	653 (43.9%)	56	37 (66.1%)	2.642 (1.488–4.692)	
Middle education	1,314	484 (36.8%)	36	17 (42.7%)	1.543 (0.787–3.025)	0.008^**^
High education	1,527	497 (32.5%)	55	15 (27.3%)	0.737 (0.396–1.373)	

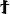
*Only suitable for participants with normal MMSE scores*.

*OR, odds ratio; CI, confidence interval; MoCA, Montreal Cognitive Assessment; MCI, mild cognitive impairment; pRBD, probable rapid eye movement sleep behavior disorder; MMSE, Mini-Mental State Examination*.

† *Adjusted for age, gender, hypertension, diabetes, hyperlipemia, stroke, depression for cognitive impairment and MCI; adjusted for age, hypertension, diabetes, hyperlipemia, stroke, depression, MMSE score for decreased gait speed*.

Significance was set at p = 0.05. Significant differences are highlighted by

** p < 0.01 and

*** *p < 0.001*.

## Discussion

Our results suggest that pRBD is associated with cognitive impairment and gait dysfunction among community-dwelling older people. This relationship between pRBD and cognitive/motor function is potentially modified by an individual's educational level.

The association between RBD, cognitive deficits, and motor abnormalities has been well-recognized, but previous studies mostly focused on PSG-confirmed RBD cases in clinical settings (25–27). However, RBD patients in clinical settings could have a more severe form of RBD, hence could present entirely different characteristics from the general population, which has been least studied. Our findings provide additional evidence on the association based on a questionnaire-screened pRBD in the general population. A previous study conducted by Boot et al. (7) reported an over 2-fold increased risk of developing MCI in pRBD subjects with a normal baseline cognition over a 4 year follow-up. In our study, pRBD was not associated with MCI measured by MoCA and MMSE scores but was associated with an ~2-fold increased risk of developing cognitive impairment measured by MMSE. This discrepancy may be explained by the low sensitivity of MoCA and MMSE in identifying MCI in pRBD. A previous community-based study conducted by McDade et al. (9) showed that participants with pRBD had subtle changes in gait characterized by decreased velocity and cadence and increased stride-to-stride variability with an automated gait analysis system. We now have confirmed the association between pRBD and gait abnormalities in a much larger community population.

Our findings show that the effect of pRBD on MCI is influenced by the level of education. The moderating effect of education was more prominent when MCI was assessed by MoCA score than MMSE score. pRBD was associated with an increased risk of MCI in participants with a low level of education. Interestingly, a decreased risk of MCI was seen in participants with a high level of education with pRBD. Consistent with our findings, a meta-analysis conducted in PD showed higher education to be associated with better cognitive performance (14). However, the long-term diagnosis of dementia and the rate of cognitive decline in PD were not affected by educational attainment (14, 15). In fact, the cognitive reserve hypothesis proposed that education may protect against the onset of disease-related clinical symptoms (28). Our findings support the cognitive reserve hypothesis in the prodromal stage of PD showing high education to protect against MCI associated with pRBD. However, the interaction effect between education and pRBD on cognitive impairment did not reach statistical significance. This difference may suggest that the beneficial effect of education is more prominent in the early stage of cognitive decline. Similar effects have been reported in aging and Alzheimer's disease (AD) studies (14, 29, 30). A study by Groot et al. (30) compared the effect size of education according to the disease stage of AD, showing that education had greater effects on attention and executive function in the pre-dementia state than in dementia. Nevertheless, the effect size of education in different stages of α-synucleinopathy and domain-specific effects yet remain to be investigated.

The association between education and motor function has also been investigated in healthy older adults and PD patients. Community-dwelling older persons with less education had slower walking speed and were more susceptible to motor dysfunction caused by white matter lesions (10). In cross-sectional studies, PD patients with higher education exhibited fewer motor impairments but greater dopamine reductions in the posterior putamen, indicating that higher education provides greater levels of compensations to cope with dopamine deficits (31, 32). In a longitudinal study, education was associated with delayed emergence of Hoehn and Yahr stage ≥3, but not with the rate of motor decline in PD (15). Our findings extend the past findings by showing the protective effect of education on gait speed in the early stage of disease when the motor symptoms have not yet been manifested. Furthermore, rather than examining the direct relationship between education and motor function in PD or healthy older adults, we investigated the interaction effect between education and pRBD in the context of a community-dwelling population.

Experimental evidence suggests that education influences the locus coeruleus–noradrenergic (LC/NA) activity including anti-inflammatory, stimulating endogenous neurotrophic factors, promoting neurogenesis, and reducing oxidative stress (33). The LC/NA system plays an essential role not only in the cognitive process (34) and motor manifestations (35) but also in modulating REM sleep (36, 37). Severe neuronal loss and gliosis in the LC were observed in patients with RBD (38), and neuroimaging findings suggested that noradrenergic deficits might also contribute to cognitive impairment in RBD patients (39). We suspect that the LC may be the important structure where education plays a role in RBD.

Some of the limitations of our study need to be considered. First, this is a cross-sectional study, therefore, the causality between pRBD, education, and cognitive/motor function cannot be confirmed. It is crucial to perform prospective studies to better understand the different trajectories of cognitive/motor decline between RBD subjects with different levels of education. Second, pRBD was diagnosed by a questionnaire without PSG confirmation, which may lead to both false-positive and false-negative results (40, 41). For instance, the prevalence of pRBD (i.e., 3.3%) in our study is approximately similar to those of other community-based studies (i.e., 2.7–5.9%) (42–44) but is higher than those in PSG-confirmed RBD studies (i.e.,0.74–1.15%) (40, 45, 46). Misclassification of participants with pRBD may attenuate the significance of our findings. Third, assessments of cognitive functions were limited to MMSE and MoCA, and motor function was limited to walking speed. Hence, the detailed neuropsychological and motor characteristics of the participants could not be identified.

Despite these limitations, our study has several strengths. We evaluated the relationship between pRBD and cognitive/motor function, as well as the moderating effect of education in a relatively large community-based population. To our knowledge, this is the first study to investigate the moderating effect of education and explore the cognitive reserve hypothesis in pRBD. In addition, previous studies mostly focused on the effect of education on cognitive function, which was often confounded with a premorbid intellectual level. We extend the previous findings by showing the beneficial effect of education equally on motor function, which may provide insights into the motor system compensation in pRBD.

In conclusion, we found a close relationship between pRBD and cognitive/motor impairment. A high educational level may alleviate the detrimental effects on cognitive and motor functions caused by pRBD. Further studies are needed to confirm the protective role of education in a prospective population and elucidate the moderating mechanisms of education in RBD patients.

## Data Availability Statement

The datasets generated from this study are available on request to the corresponding author.

## Ethics Statement

The studies involving human participants were reviewed and approved by Ethics Committee of Xuanwu Hospital of Capital Medical University. The patients/participants provided their written informed consent to participate in this study.

## Author Contributions

MC contributed to the study design, manuscript preparation, and drafting. JChe, XX, SJ, and JChh contributed to the interpretation of data analysis and revising the manuscript. FQ and XW contributed to statistical analysis. ZG and PC contributed to study supervision and coordination.

### Conflict of Interest

The authors declare that the research was conducted in the absence of any commercial or financial relationships that could be construed as a potential conflict of interest.
